# Spontaneous Restoration of the Membrana Tympani

**Published:** 1890-12-27

**Authors:** 


### SPONTANEOUS RESTORATION OF THE MEMBRANA TYMPANI.

Dr. Eitelberg, of Vienna, reports an instance of complete and almost unique recovery from grave injury to the auditory apparatus. A man carrying a heavy basket on his head let it fall, and in so doing had his head compressed between the load and the wall of the cellar. There was an immediate and profuse discharge of blood from his nose and left ear, which latter lasted all night, and was still going on when the ear was examined next day. After the ear had been cleared by syringing the membrana tympani was seen to be extensively ruptured. Politzer's inflation was performed, and the canal lightly closed with iodoform gauze. The hæmorrhage gradually diminished, but did not cease until the fifth day, when the semi-detached piece of the membrane became loose and was removed by syringing. It measured 6 mm. by 4 mm. The head of the stapes could be well seen, but the malleus was drawn back by the tensor muscle. In eight weeks the membrane was completely restored, and the hearing was so good that a whisper could be heard at a distance of nearly four feet.

